# Refabrication of a Removable Partial Prosthesis Using a 3D-Printed Record Base for a Patient With Microstomia

**DOI:** 10.7759/cureus.98270

**Published:** 2025-12-01

**Authors:** Mariko Hattori, Manjin Zhang, Yuka Sumita, Noriyuki Wakabayashi

**Affiliations:** 1 Advanced Prosthodontics, Institute of Science Tokyo, Tokyo, JPN; 2 Stomatological Hospital, School of Stomatology, Southern Medical University, Guangdong, CHN; 3 Partial and Complete Dentures, The Nippon Dental University, School of Life Dentistry at Tokyo, Tokyo, JPN

**Keywords:** 3d printing, digital dentistry, maxillofacial prosthetics, microstomia, removable partial denture

## Abstract

Microstomia, which is characterized by reduced oral opening, poses significant challenges in prosthesis fabrication, particularly during impression-taking. This technical report describes a novel workflow combining conventional techniques with digital technology to refabricate a removable partial denture for a 65-year-old man with microstomia. The process utilized 3D scan data of the patient’s previous prosthesis to create a 3D-printed record base, eliminating the need for full-arch impressions and reducing the burden on both the patient and practitioner. The transparent printed base enabled precise adjustments, and conventional techniques were employed for clasp fabrication and functional seating impressions. This mixed workflow minimized dependency on advanced technical skills or specialized software, streamlining the process and achieving favorable clinical outcomes. The approach demonstrates the potential of integrating digital technology with traditional methods to address the unique challenges of prosthesis fabrication for patients with microstomia.

## Introduction

Microstomia, which is defined as reduced opening of the mouth, can occur as a result of congenital anomalies, burns, cancer resection, and conditions such as Stevens-Johnson syndrome [1,2]. Fabricating prostheses for patients with microstomia poses various challenges, particularly in impression-taking [3,4]. After the completion of the prosthesis, frequent adjustments are often needed to accommodate the complex defect morphology, and several relining or extensions of the denture base may be required in the oral cavity until a functional form is achieved. Recently, digital technology has been increasingly applied in the production of prosthetics [5,6]. Previous studies have evaluated the efficiency of using digital technology in prosthesis fabrication. However, it can be difficult to insert the head of an intraoral scanner into the mouth of patients with severe trismus or microstomia [7,8]. This report presents a technique that applies digital technology to the refabrication process of a removable prosthesis for patients with microstomia, thereby reducing the burden on both the patient and the practitioner.

A 65-year-old man with microstomia due to scar healing of multiple oral mucosal ulcers experienced difficulty with a fabricated denture. Ten years earlier, he noticed ulcers in the oral cavity and visited the oral surgery department of the university hospital. The patient underwent a thorough examination for suspected conditions, including cancer, but all were ruled out, and the cause of the ulcers remained unknown. The ulcers spread to the pharynx and larynx, necessitating a tracheostomy. His premolars and molars were missing, but limited treatment, such as conservative procedures on the incisors, was possible. Several Kampo formulas, including Kakkonto (TJ-1, Tsumura, Tokyo, Japan), Shosaikoto (TJ-9, Tsumura), Shoseiryuto (TJ-19, Tsumura), and Sanoshashinto (TJ-113, Tsumura), were prescribed by his primary care physician. Of these, Sanoshashinto (TJ-113, Tsumura) showed remarkable efficacy. After receiving Kampo therapy, the ulcers went into remission, allowing for removable prosthetic treatment. Fabrication of a prosthesis in a single step was challenging because of microstomia resulting from the healing of lip ulcers. Therefore, a smaller denture was initially fabricated using conventional methods and later adjusted intraorally with auto-polymerizing acrylic resin to extend the base. After six years of use, a new denture was recommended due to material deterioration caused by repeated adjustments and repairs; however, his microstomia had become even more severe. As insertion of the impression tray into the oral cavity was difficult, a method for fabricating a new denture based on the 3D surface data of the existing denture was planned and implemented, as described below.

## Technical report

**Figure 1:**
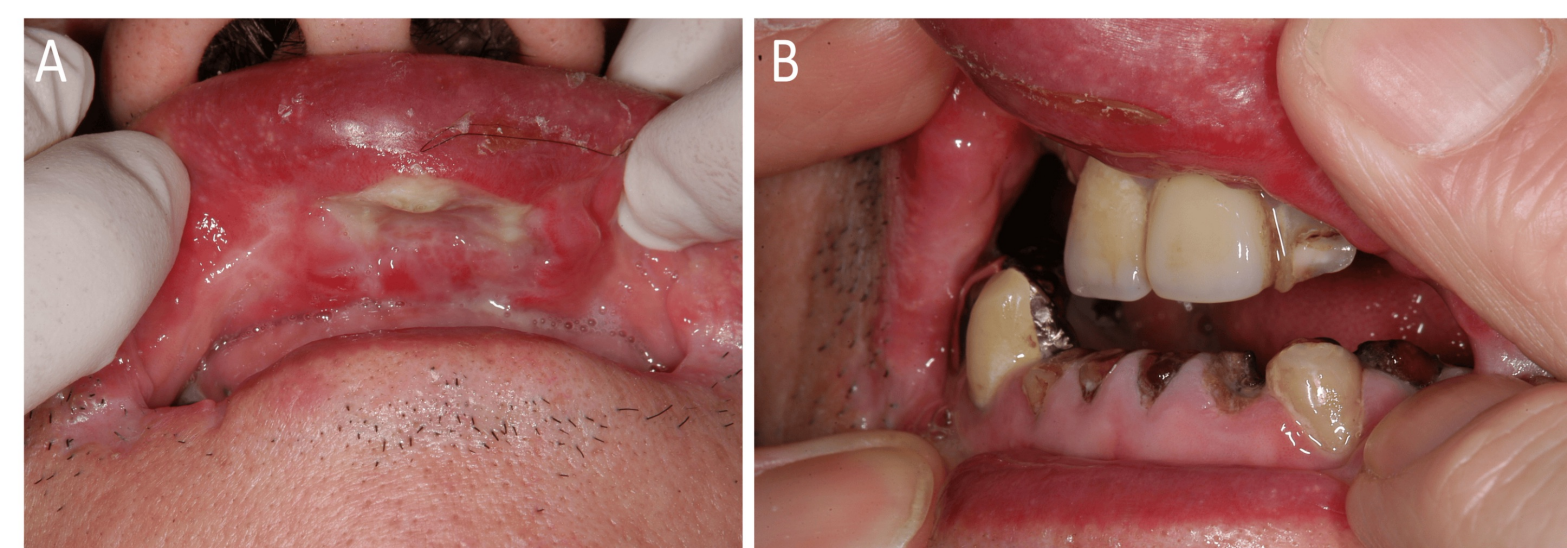
Patient’s condition at the first visit A: ulcer on the upper lip; B: maximum mouth opening with the aid of the patient’s own fingers

The technique used to refabricate a maxillary prosthesis for a patient with microstomia (Figure 1) is described below.

**Figure 2:**
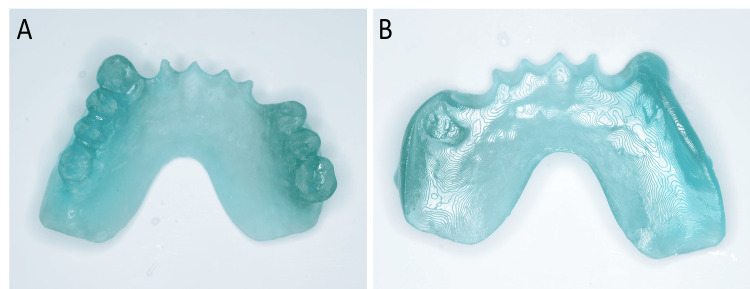
3D print of the previous prosthesis using a UV-curable material, utilized as an occlusal record base A: occlusal view; B: intaglio view

**Figure 3:**
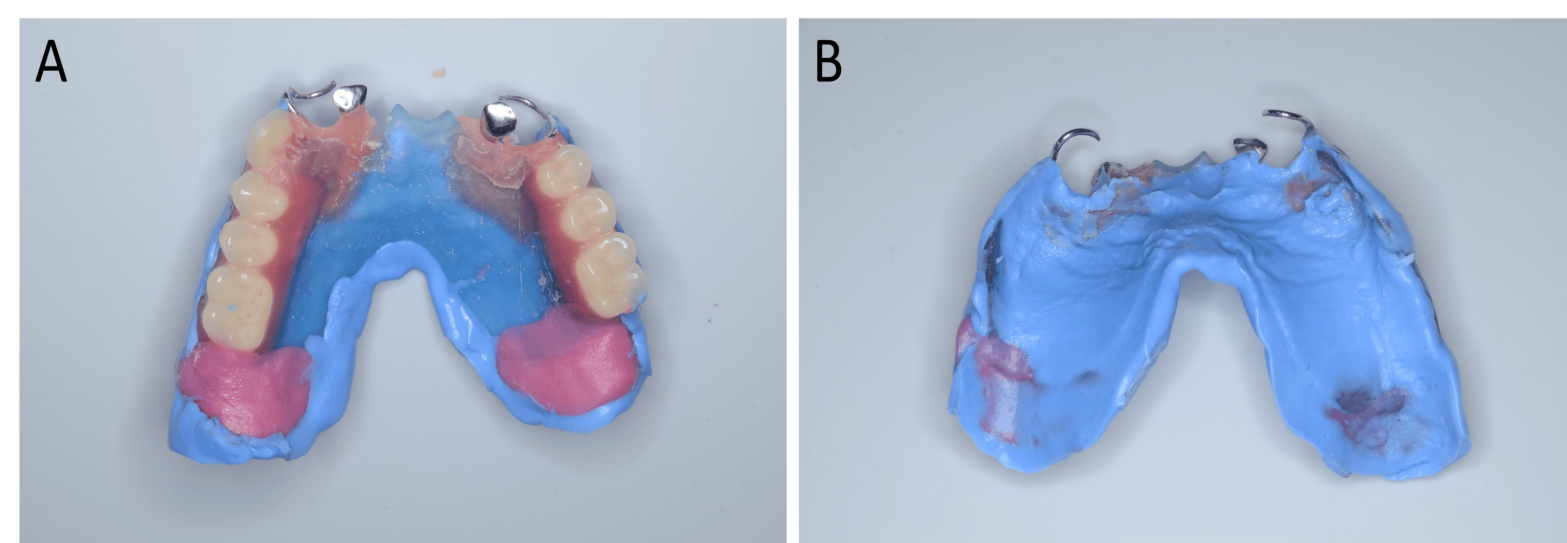
Functional seating impression using a wax trial denture A: occlusal view; B: intaglio view

**Figure 4:**
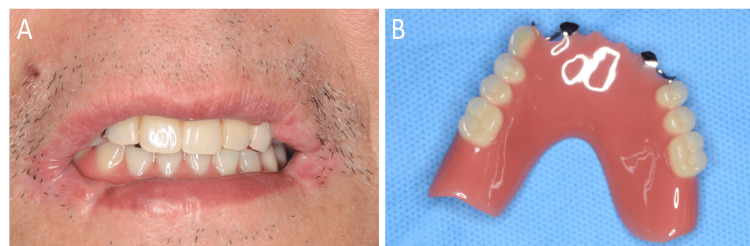
Completed prosthesis A: intraoral view with the prosthesis in place; B: occlusal view

1. Make a digital impression of a partial prosthesis with an intraoral scanner (Trios3, 3Shape, Copenhagen, Denmark) and obtain 3D surface data.

2. Make a conventional impression of the anterior abutment teeth, using a small sectional stock tray (Rotating Tray Fusayama Small, YDM, Tokyo, Japan) and an irreversible hydrocolloid material (Algiace Z, Sankin, Tokyo, Japan).

3. Print out the 3D data with a UV-curable material composed primarily of methacrylate ester (Dental SG, Formlabs, Somerville, MA, USA) using a 3D printer (Form3, Formlabs) (Figure 2).

4. Use the printed model as an occlusal record base, adjust intraorally, and make the occlusal registration. It is possible to extend at this stage using an impression compound (Iso Compound, GC, Tokyo, Japan).

5. Remove the teeth part of the base, using a carbide bur, and align the artificial teeth (Endura Posterio, Shofu, Kyoto, Japan) with wax (Paraffin Wax, GC) and make a wax try-in denture.

6. Fabricate the retainers, each consisting of a bent cobalt-chromium wire and a cast cobalt-chromium alloy rest, on the working model obtained from the conventional impression described in technique #2.

7. Try the wax denture, fix the retainers to the 3D-printed base intraorally using self-curing acrylic resin (Unifast III, GC), and make the functional seating impression with a hydrophilic vinyl polysiloxane impression material (Examixfine Regular Type, GC) (Figure 3).

8. Process it with heat-polymerized acrylic resin (Acron Dark Pink, GC) and then deliver the prosthesis (Figure 4).

## Discussion

Using the 3D data of the old prosthesis as an occlusal rim enabled us to omit both the preliminary and final impression steps. The printing materials were transparent, making them suitable for confirming the fit of the occlusal record base’s mucosal surface. In the present case, the clasps were fabricated by taking conventional impressions of the abutment teeth with a small rotational tray and attaching them to the denture intraorally. At this stage, using an intraoral scanner for the remaining teeth might also be an option, given that the digital impressions of several teeth are feasible in some microstomia cases, depending on the severity of the trismus [7,8]. However, digital workflows that require superimposing intraoral scans, prosthesis scans, and model scans require specialized software and techniques. Designing a prosthesis with 3D software additionally requires the expertise of a skilled dental technician. Thus, a workflow that incorporates more digital processes becomes increasingly dependent on the technician’s proficiency.

In contrast, the method used in this case was a hybrid of conventional and digital approaches and was less technician-dependent because it only required printing the denture data with a 3D printer without design modification. Moreover, the seating impression compensated for potential inaccuracies of 3D printing, including layer lines in the printing material. The final form of the denture, which had been gradually refined through long-term adjustments of the old prosthesis, was thereby transferred to the new denture, resulting in a favorable outcome.

In this patient, a complete denture was fabricated for the mandible using a similar but simplified method because no retainer was necessary. The dentures for both jaws demonstrated good fit and function, requiring fewer adjustments compared with the previous dentures. This report illustrates that, in challenging microstomia cases, dentures can be fabricated using 3D scan data of old prostheses without the need for full-arch trays or full-arch digital impressions, which are often difficult to insert in patients with microstomia [1,7,8].

The main limitation of this technique is the requirement for a previous denture. When no previous denture is available, recording the mucosal surface with soft materials, such as putty-type impression material or modeling compound, is another potential approach [1]. If a soft material that can expand intraorally is used and subsequently digitized extraorally, it may provide a simpler alternative for capturing the prosthesis surface in patients with microstomia. In addition, the use of impression materials for clasp fabrication becomes problematic when microstomia is too severe to permit the insertion of even the smallest rotational tray. In such cases, wire clasps can be fabricated and adjusted chairside without impressions. Special clasp designs, such as ball clasps, provide greater flexibility for chairside modification. The use of an intraoral scanner with a small scanner tip could also be a solution. Another limitation of this technique is the intraoral fixation of the retainer, which carries a risk of aspiration or swallowing. Attaching dental floss to the retainer could be a simple and effective option to prevent such incidents.

## Conclusions

This report detailed a workflow combining conventional techniques with digital technology, thereby enabling the fabrication of dentures for a patient with microstomia. By utilizing 3D scan data of the old prosthesis, the process was simplified, reducing the need for full-arch impressions. The mixed workflow combining conventional techniques with digital technology allowed for a simplified process that was less dependent on advanced technical skills or specialized software, resulting in a favorable clinical outcome.
